# Transient oxygen-rich air from air outlets promptly detected by failure of end-tidal control anesthesia

**DOI:** 10.1186/s40981-018-0201-2

**Published:** 2018-09-04

**Authors:** Takashi Suzuki, Naoki Otsuka, Yuuya Kohzuka, Shin-Ya Kimura, Sayaka Ohara

**Affiliations:** 1 Department of Anesthesiology, Showa University Koto Toyosu Hospital, 5-1-38, Toyosu, Koto-ku, Tokyo, 135-8577 Japan; 2 Department of Anesthesiology, Showa University Fujigaoka Hospital, 1-30, Fujigaoka, Aoba-ku, Yokohama, 227-8501 Japan; 3 Division of Anesthesiology, Department of Perioperative Medicine, Showa University School of Dentistry, 2-1-1, Kitasenzoku, Ohta-ku, Tokyo, 145-8515 Japan

**Keywords:** Air, Oxygen, Gas blender, Contamination, Piping system, End-tidal control, Fresh gas control

## To the Editor,

We occasionally experienced abrupt spontaneous changes from end-tidal control (EtC) to fresh gas control mode accompanied by a beep during anesthesia with an air/oxygen mixture using a GE Aisys Anesthesia Carestation™ (GE Healthcare Japan Co., Ltd., Hino, Japan) [1]. When the EtC mode was aborted, both the Aisys and patient monitor displayed unusually high inspired oxygen concentrations that did not correspond to the flowmeter readings of the Aisys, the situation usually persisting for several minutes. This indicated a surge in the inspired oxygen concentration despite no change in fresh gas flow settings. The phenomenon was observed only on the Aisys used in the three adjacent rooms in our operating room suite. Air outlets installed in these three rooms originate from a common upper level and are located at the same hierarchy level in the central pipeline system.

**Fig. 1:**
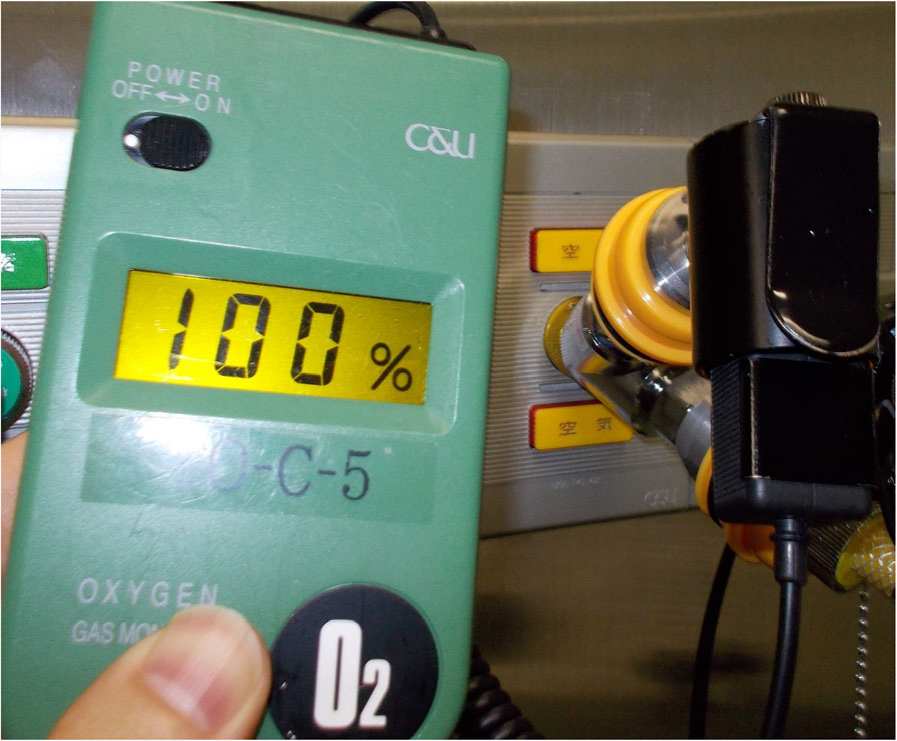
Oxygen-rich air of up to 100% oxygen was occasionally detected by a handheld oximeter (XO326-BC, New Cosmos Electric, Osaka, Japan) connected to medical air outlets in certain operating rooms

With frequent checks on the oxygen concentration at our air outlets, increases of up to 100% oxygen lasting a few minutes were occasionally detected in those rooms using a handheld oxygen analyzer (XO326-BC, New Cosmos Electric Co., Ltd., Osaka, Japan) (Fig. 1). To exclude the possibility of oxygen reflux toward the air pipeline within the Aisys, the air source for the Aisys was changed from the pipeline to stand-alone air compressors. No EtC failure occurred during this investigation period, although oxygen-rich air from air outlets in the three rooms was still observed.

In one of the three operating rooms, a brand-new infant warmer (Infa Warmer i™; Atom Medical Co., Ltd., Tokyo, Japan) for babies born by cesarean section was present. Both the oxygen and the air inlet of the warmer were always connected to the pipelines at that time. We speculated that the phenomenon was caused by the intermittent supply of oxygen-rich air into the air inlets of the Aisys, which resulted in failure of closed-loop control of the end-tidal oxygen concentration. When we tested our hypothesis by disconnecting the pipelines to the warmer when not in use, the phenomenon disappeared but reappeared after restoring the connection.

Based on the circumstantial evidence, we requested that the manufacturer investigate whether the blender could function as a conduit between air and oxygen pipelines. The warmer manufacturer and built-in blender supplier (Bio-Med Devices Inc., Guilford, CT, USA) initially denied any fault with their systems, but later, after repeated requests for further investigation, admitted that the product was defective. In multiple experiments performed, the oxygen-rich air was replicated when the blender that had been used in the operating room was in an idle state. The manufacturer’s report concluded that oxygen reflux within the blender could not be prevented completely.

Blood gas analysis results with unexpectedly high oxygen tensions or alarm detection of unintended high inspired oxygen concentration have been reported as triggers for the discovery of faulty blenders built into ventilators used in intensive care units [2–4]. In routine anesthesia, the upper alarm limits for inspired oxygen concentration are frequently turned off. Therefore, transient oxygen-rich air can be easily overlooked.

To summarize, we reported oxygen-rich air supplied sporadically and transiently from pipelines, which was promptly detected by EtC failure. The cause was a faulty air/oxygen blender built into an infant warmer installed in one of our operating rooms. Our experience suggests that air/oxygen blenders should not be left connected to pipelines when not in use. If they are left connected, the bleed switch on the warmer should be turned on, allowing partial release of the air/oxygen from the blender into the atmosphere, preventing cross-contamination.

## Abbreviation

EtC: End-tidal control
